# Host plants of the non‐swarming edible bush cricket *Ruspolia differens*

**DOI:** 10.1002/ece3.5016

**Published:** 2019-02-28

**Authors:** Robert Opoke, Philip Nyeko, Geoffrey M. Malinga, Karlmax Rutaro, Heikki Roininen, Anu Valtonen

**Affiliations:** ^1^ Department of Biology Gulu University Gulu Uganda; ^2^ Department of Environmental and Biological Sciences University of Eastern Finland Joensuu Finland; ^3^ Department of Forestry, Biodiversity and Tourism Makerere University Kampala Uganda

**Keywords:** edible insect, feeding ecology, nsenene, tropical grasslands, Uganda

## Abstract

The edible *Ruspolia differens* (Orthoptera: Tettigoniidae) is a widely‐consumed insect in East Africa but surprisingly little is known of its host plant use in the field. We studied host plants used by non‐swarming *R. differens* for 15 months, in central Uganda. In particular, we assessed the use of host plant species with respect to host cover in the field and host parts used by *R. differens*, also recording their sex, developmental stages, and colour morph. *Ruspolia differens* were found on 19 grass and two sedge species and they were observed predominantly (99% of 20,915 observations) on seven grasses (namely, *Panicum maximum*, *Brachiaria ruziziensis*, *Chloris gayana*, *Hyparrhenia rufa*, *Cynodon dactylon*, *Sporobolus pyramidalis*, and *Pennisetum purpureum*). *Ruspolia differens* was most frequently observed on the most common grass of each study site but *P. maximum*, and *S. pyramidalis* were used more frequently than expected from their cover in the field. Furthermore, *R. differens* were observed predominantly on inflorescences (97% of feeding observations) and much less frequently on the leaves (3.0%), stems (0.1%), and inflorescence stalks (0.1%) of grasses and sedges. Host use was not independent of sex, developmental stage, or colour morph. *Panicum maximum* was the preferred host of the youngest nymphs of *R. differens*. Overall, our findings indicate that a continuous supply of diverse grass resources with inflorescences is necessary for the management and conservation of wild populations of *R. differens*.

## 1 INTRODUCTION

**Figure 1:**
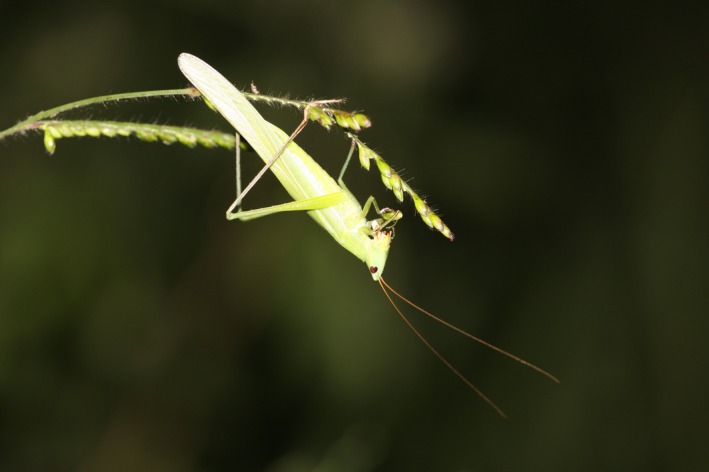
*Ruspolia differens*, green colour morph, feeding on *Brachiaria ruziziensis*, at Kabanyolo, Uganda. Photo: A. Valtonen (8 May 2016)

Edible insects represent an important source of food and livelihood for humans, particularly in developing countries (van Huis et al., 2013; Kelemu et al., 2015; Okia et al., 2017). They could provide a sustainable, cost‐effective, and high‐quality alternative source of protein and fatty acids to traditional livestock products (van Huis et al., 2013). This fascinating potential has generated unprecedented global interest in edible insects over the last few years (van Huis & Tomberlin, 2017). The edible *Ruspolia differens* Serville (Orthoptera: Tettigoniidae), known locally in Uganda as “nsenene”, is a highly‐prized and widely‐consumed edible insect in East Africa (Okia et al., 2017; Figure 1). It occurs in swarming and non‐swarming phases, and in eight colour morphs, in tropical Africa and some islands of the Indian Ocean (Bailey & McCrae, 1978; Massa, 2015). The typical colour morphs are green and brown, while rarer colour morphs include purple superimposed on either green or brown (Bailey & McCrae, 1978; McCrae, 1982). In non‐swarming populations, all developmental stages can be found throughout the year but the population densities are higher in the rainy seasons and lower in the dry seasons (Opoke et al., 2018). The individuals are mainly nocturnal, but in the swarming phase the activity‐level can remain high during the daytime, when they can opportunistically be predated by birds (Bailey & McCrae, 1978). Swarming usually occurs during and after the peak of the rainy seasons, for example, in Uganda, around May and in November–December (Bailey & McCrae, 1978). Wild *R. differens* are harvested during swarming periods with increasingly efficient light traps (Okia et al., 2017), yet the specific reproductive areas of the swarming *R. differens* have not been identified, and the long‐term consequences of harvesting wild populations are completely unknown.

Surprisingly little is known about host plants used by *R. differens*. In the field, individuals have been observed feeding mainly on flowers and the young grains of grasses (Bailey & McCrae, 1978; Swaine, 1964). When reared in the laboratory they readily accept artificial foods and the leaves, flowers, and grains of many grasses, including cultivated cereals (Brits & Thornton, 1981; Hartley, 1967; Malinga et al., 2018a, 2018b; Valtonen et al., 2018). Most accounts of *R. differens*’ requirements are based on sporadic field observations, fragmentary data, or are deduced from experience in the laboratory. Empirical information on the host plants used by *R. differens* in the wild is needed to understand the importance of different plant species for the nutritional ecology of *R. differens*. Such information could be also used in future for the management and conservation of *R. differens *populations in the wild.

In this study, we report a long‐term assessment of the host plants used by non‐swarming *R. differens* in two semi‐natural grasslands of central Uganda. We addressed the following questions: (a) What plant species do *R. differens* use in the field, and are the plant species used in the same proportions as they occur in the field? (b) What plant parts are used by *R. differens* in the field? (c) Do females and males, or different developmental stages, or different colour morphs of *R. differens* differ in their host use? Overall, are (d) females and males, and (e) the green and brown colour morphs represented in equal proportions in the non‐swarming population? If *R. differens* is a facultatively oligophagous grass‐specialist, as predicted by laboratory assessments, with a clear preference for certain grass or sedge species (Valtonen et al., 2018), we predicted that certain host species are used more frequently than their abundance in the field alone would predict. We also hypothesised that host use by *R. differens *may vary depending on sex or developmental stage, due to the different physiological state or nutritional requirements of individuals (Behmer & Joern, 1994; Boys, 1978; Unsicker, Oswald, Köhler, & Weisser, 2008). Finally, we hypothesised that host use is different among colour morphs since different grasses potentially provide different levels of camouflage for green and brown colour morphs (Karpestam & Forsman, 2011).

## 2 MATERIALS AND METHODS

### 2.1 Study area and sites

The study was conducted from November 2015 to January 2017 in grazing lands at the Makerere University Agricultural Research Institute, Kabanyolo (MUARIK), Uganda. This mixed farm is located at 0°28′N, 32°37′E, about 20 km north of Kampala, at an elevation of approximately 1,150 m a.s.l. Rainfall patterns are bimodal, most rain occurs between March and May, and between September and November, with mean annual rainfall of 1,170 mm (Nsubuga, Olwoch, & Rautenbach, 2011). The mean daily minimum temperature in the Kampala region is 17.6°C, and the mean daily maximum temperature is 27.8°C (WMO, 2018).

**Figure 2:**
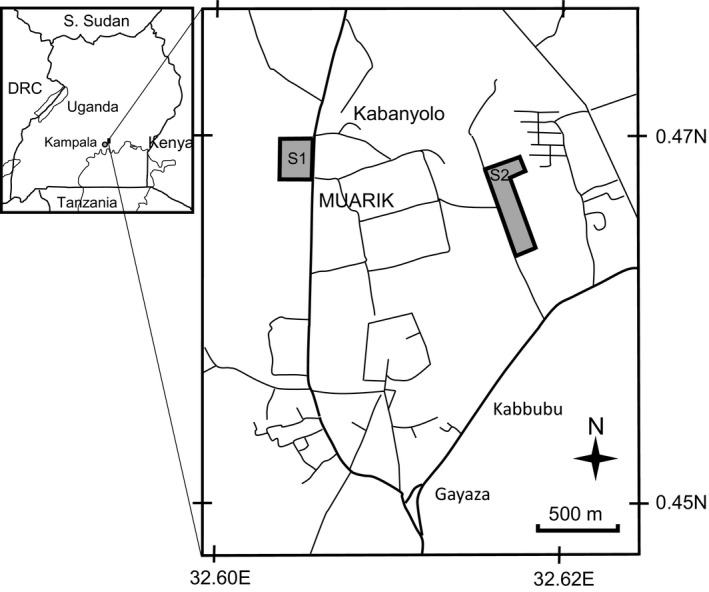
Location of the study. Site 1 (S1) and Site 2 (S2) at the Makerere University Agricultural Research Institute, Kabanylo (MUARIK), Uganda

Two study sites were selected at MUARIK (Figure 2). Site 1 was 5.2 ha and located 1.5 km from Site 2, which measured 11.0 ha. The study sites were open grasslands dotted with trees, shrubs, and herbs. The eastern side of Site 1 was dominated by Elephant grass, *Pennisetum purpureum *Schumach. Cassia trees, *Senna spectabilis *(DC.) H.S.Irwin & Barneby, the shrubs *Flueggea virosa* (Willd.) Voigt, and *Phytolacca dodecandra* L'Hér., and herbs *Elephantopus scaber* L. var. *brevisetus* Philipson were scattered throughout the field. At Site 2, *Vernonia amygdalina* Delile, *Vernonia auriculifera *Hiern, *Stachytarpheta urticifolia* Sims, and *Lantana camara* L., and herbs, e.g., *Indigofera arrecta* Hochst. ex A.Rich. and *Elephantopus scaber* L. var. *brevisetus* Philipson were scattered throughout the eastern side which was dominated by Guinea grass, *Panicum maximum* Jacq., Rhodes grass, *Chloris gayana* Kunth, and Spear grass, *Imperata cylindrica* (L.) P.Beauv. At both study sites, the western sides were dominated by Guinea grass, *Panicum maximum *Jacq., Rhodes grass, *Chloris gayana* Kunth, Thatch grass, *Hyparrhenia rufa *(Nees) Stapf, Congo signal grass, *Brachiaria ruziziensis *R.Germ. & C.M.Evrard, and sedges, e.g., *Kyllinga elatior* Kunth, *Cyperus distans* L.f. and *Paspalum scrobiculatum* L.

### 2.2 Use of host plants

Data on the host plants used by *R. differens* was collected by walking along a pre‐defined trail, established at both study sites, and starting at a random point on the trail at the beginning of each census. On each sampling night the study site was censused for 3.5 hr between 7:30 and 11:00 p.m. All observations were made by the same observer (R.O.) using binoculars and an overhead light. Each study site was visited between one and six nights per month (in 83% of the cases between three and five nights per month) for over 15 months between November 2015 and January 2017. Whenever *R. differens *was spotted, the plant species, and the sex, developmental stage, and colour morph of the insect were recorded. For the individuals which were observed feeding (67% of total observations), the plant part (inflorescence, leaf, stem, or inflorescence stalk) being eaten was also recorded. Species identification of host plants was ensured by collecting samples of plants and identifying them at the herbarium of Makerere University following Clayton, Phillips, and Renvoize (1974) and Katende, Birnie, and Tengnäs (1995). The sex of individuals was only determined starting from 4th instar because these stages have developed sex characteristics (the presence of ovipositor can be used to identify females). Developmental stages were categorised as 1st to 6th instars for males and 1st to 7th instars for females (only females have a 7th instar) depending on body length (Brits & Thornton, 1981). The developmental stages were later pooled as “small nymphs” (stages 1–3), “medium nymphs” (stages 4 and 5), “large nymphs” (stages 6 and 7), and “adults”. Furthermore, the colour morph of each individual was categorised as either “green” or “brown” for statistical analysis.

### 2.3 Host plant cover and number of inflorescences

To determine the availability of host plants in the field, the leaf cover and number of inflorescence of grasses and sedges (the potential host plants) were measured at both study sites during the peak of the first (April–May) and second (November–December) annual rainy seasons in 2016. We established parallel, 150 m long transects at distances of 50 m at both study sites. Along each transect line, 1.8 m radius circular plots were established every 50 m. Whenever the plot extended into trees, shrubs, or herbs only, the plot was relocated by moving it 10–15 m forward into grasslands (thickets of shrubs and trees were excluded because *R. differens* are mostly grassland dwellers; Bailey & McCrae, 1978). This method produced a total of 58 plots (24 plots at Site 1 and 34 plots at Site 2).

Each plot was divided, through its centre, into four equal sections. In each section, the percentage leaf cover of every grass and sedge species encountered (16 species) and herbs (pooled) and trees (pooled) was visually scored using the scale: 0%, 1%, 2%, 3%, 4%, 5%, 6%, 7%, 8%, 9%, 10%, 15%, 20%, 25%, …, 95%, 100%. Furthermore, the number of inflorescences of all grass and sedge species was counted. To minimise the risk of subjective errors when estimating leaf cover, the same person (R.O.) conducted the estimates in both the study sites. For each plot, we calculated the average of leaf cover (or number of inflorescences) across the four sections and across studied times and used these values in statistical analysis.

### 2.4 Statistical analysis

We decided to use only leaf cover as an indicator of host plant availability in the statistical analysis, because we considered it to be a more robust estimator of host‐plant availability (as different grasses can flower at slightly different times of the year, and also because inflorescences can be eaten very fast). However, the leaf cover and number of inflorescences of plant species correlated strongly and positively with each other across the studied plots indicating that leaf cover is a good indicator of availability of inflorescences. The correlations were calculated for all observed grass and sedge species with sufficient data (i.e., 13 species; excluding *Eleusine africana,* *Setaria homonyma *and *Stenotaphrum dimidiatum*, for which the data was not sufficient; Spearman's rho, mean across species = 0.77; min = 0.41; max = 0.98; for each species *N* = 58).

Chi‐square goodness‐of‐fit tests (for the two study sites separately) were conducted in order to test if the grass and sedge species are used by *R. differens* in the same proportions as they occur in the field. For this, we calculated the observed frequencies for each of the seven most commonly used grass and sedge species, as well as for the rest of the grass and sedge species (categorised as “Rare combined”). The observed frequencies were compared to the proportions of these species in leaf cover data (summing to 1); assuming these species (and not herbs and trees) form the available resource pool for *R. differens*. We decided to keep the analysis of the two sites separate because they had different plant community compositions.

We fitted a generalized linear model (multinomial probability distribution) to analyse if host use by *R. differens* is explained by sex, developmental stage, study site, or their two‐way interactions. A second generalized linear model (with multinomial probability distribution) was fitted to analyse if host use is explained by developmental stage, colour morph, study site, or their two‐way interactions. We fitted these models separately because the sex of individuals could only be determined starting from 4th instar onwards and therefore the first model was based only on a subset of data.

Finally, Chi‐squared goodness‐of‐fit tests, separately for the two study sites, were conducted to test if female and male, or green and brown, *R. differens* occurred in equal proportions. Statistical analysis was performed using R version 3.3.2 (R Core Team, 2014) and SPSS version 23 (Armonk, NY, USA).

## 3 RESULTS

### 3.1 Host plants and their parts

**Figure 3:**
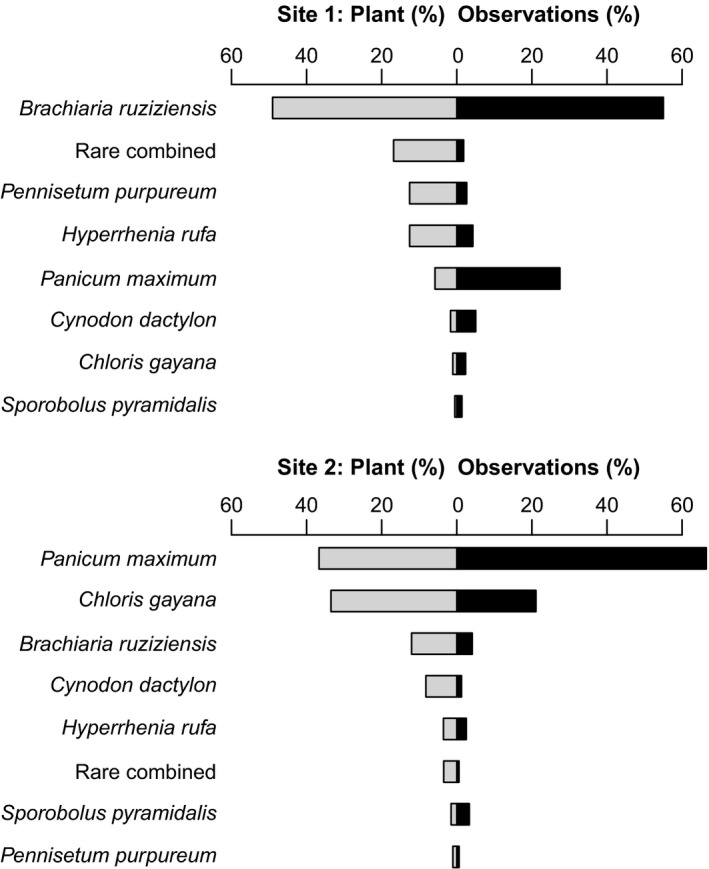
The proportion of observations on the seven most commonly used host plants (right) versus their proportion of cover for all grasses and sedges in the field (left); the rest of the grasses and sedges pooled as “Rare combined”. Plant (%) indicates the proportion of cover from all observed grasses and sedges in the study plots (summing to 100%). Observations (%) indicate the proportion of *R. differens* observations on each grass and sedge (summing to 100%)

Out of 21,252 *R. differens* observations, 20,915 (98%) were on grasses and sedges (for the remainder of the results, only observations on grasses and sedges are included). *Ruspolia differens* was found on a total of 19 grass (*Poaceae*) and two sedge (*Cyperaceae*) species (Table 1). They were observed predominantly (99% of 20,915 observations) on seven grasses, namely; *P. maximum*, *B. ruziziensis*, *C. gayana*, *H. rufa*, *Cynodon dactylon*, *Sporobolus pyramidalis *and *P. purpureum *(Table 1). At both study sites, *R. differens* were most frequently observed on the most common grass at the site (Figure 3). However, the grasses were not used in the same proportions as they occurred in the field (Chi‐square goodness‐of‐fit tests; Site 1: χ^2^ = 13,284, *df* = 7, *p* < 0.001; Site 2: χ^2^ = 4,335, *df* = 7, *p* < 0.001). At both study sites, *P. maximum*, and *S. pyramidalis* were used more frequently than expected from their leaf cover in the field (Figure 3).

**Table 1:** Mean % leaf cover of grass and sedge species, and *Ruspolia differens* (%) observed on grass and sedge species (*N* = 20,915) at MUARIK, Uganda

Host plant species	% leaf cover	% of *R. differens*
*Brachiaria ruziziensis* R.Germ. & C.M.Evrard	17.721 (±3.561)	31.494
*Panicum maximum* Jacq.	13.511 (±1.688)	45.475
*Chloris gayana* Kunth	11.150 (±2.132)	11.006
*Hyparrhenia rufa* (Nees) Stapf	4.688 (±1.735)	3.481
*Sorghum leiocladum* (Hack.) C.E.Hubb.	4.022 (±1.682)	0.091
*Pennisetum purpureum* Schumach.	3.877 (±1.699)	1.712
*Cynodon dactylon* (L.) Pers.	3.126 (±0.500)	3.299
*Sporobolus pyramidalis* P.Beauv.	0.639 (±0.151)	2.281
*Paspalum scrobiculatum* L.	0.599 (±0.140)	0.210
*Kyllinga elatior* Kunth	0.442 (±0.097)	0.053
*Cyperus distans* L.f.	0.384 (±0.082)	0.024
*Imperata cylindrica* (L.) P.Beauv.	0.194 (±0.107)	0.100
*Digitaria abyssinica* (Hochst. ex A.Rich.) Stapf	0.137 (±0.089)	0.186
*Stenotaphrum dimidiatum* (L.) Brongn.	0.065 (±0.064)	0.153
*Setaria homonyma* Chiov.	0.022 (±0.021)	0.062
*Eleusine africana* Kenn.‐O'Byrne	0.002 (±0.002)	0.134
*Setaria sphacelata* (Schumach.) Stapf & C.E.Hubb.^a^		0.206
*Setaria *sp.^a^		0.010
*Eragrostis tenuifolia* (A.Rich.) Hochst. ex Steud.^a^		0.005
*Setaria verticillata* (L.) P.Beauv.^a^		0.005
*Sporobolus consimilis *Fresen.^a^		0.014

Note

Leaf cover values are means (±*SE*) across plots at the two sites.

a Rare species not captured in the vegetation study plots, although *R. differens* was seldom found on them.

The *R. differens* were predominantly observed feeding on inflorescences (97% of 14,193 feeding observations) and much less frequently on leaves (3.0%), stems (0.1%), and inflorescence stalks (0.1%) of grasses and sedges.

### 3.2 Host plant use in relation to sex

**Figure 4:**
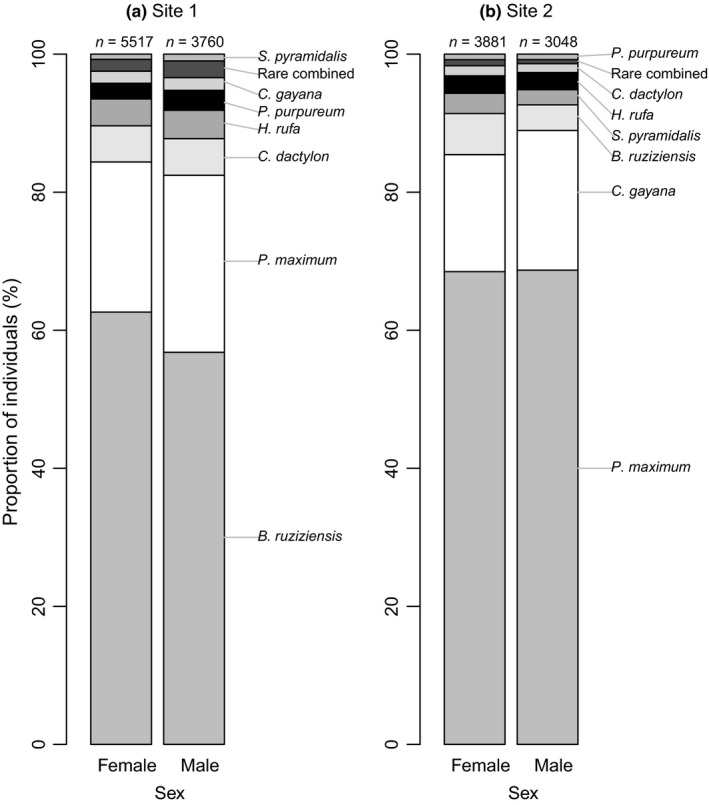
The proportion of observations on the seven most commonly used host plants (the rest of the grasses and sedges pooled as “Rare combined”) shown separately for male and female *R. differens *at (a) Site 1 and (b) Site 2

Based on the first fitted generalized linear model (including only the subset of data with information on sex of individuals), the use of host plants was explained by sex (Wald Chi‐square = 7.3, *df* = 1, *p* = 0.007), developmental stage (Wald Chi‐square = 20.4, *df* = 3, *p* < 0.001), study site (Wald Chi‐square = 3139.2, *df* = 1, *p* < 0.001), and interactions between sex and study site (Wald Chi‐square = 15.0, *df* = 1, *p* < 0.001), and developmental stage and study site (Wald Chi‐square = 67.2, *df* = 2, *p* < 0.001). This suggests that females and males used hosts differently, although this depended on the study site (Figure 4). At both sites females used *B. ruziziensis* (dominant grass at Site 1) proportionally more than males, while *P. maximum*, the dominant grass at Site 2, was used in same proportions by males and females (Figure 4). There was no interaction between sex and developmental stage (Wald Chi‐square = 0.2, *df* = 2, *p* = 0.92), indicating that the males and females of different developmental stages did not use hosts differently.

At both Site 1 (59% of observations) and Site 2 (56%), females were observed more commonly than males (Chi‐square goodness‐of‐fit tests; Site 1, χ^2^ = 332.8, *df* = 1, *p* < 0.001; Site 2, χ^2^ = 100.1, *df* = 1, *p* < 0.001). Of those developmental stages where it was possible to determine sex, females represented 49.4% of medium nymphs, 77.4% of large nymphs and 55.7% of adults (data pooled for the two study sites).

### 3.3 Host plant use in relation to developmental stage and colour morph

Based on the second fitted generalized linear model (including all observations), the use of host plants was significantly explained by developmental stage (Wald Chi‐square = 203.8, *df* = 3, *p* < 0.001), colour morph (Wald Chi‐square = 30.0, *df* = 1, *p* < 0.001), study site (Wald Chi‐square = 2581.3, *df* = 1, *p* < 0.001), and interactions between developmental stage and study site (Wald Chi‐square = 512.5, *df* = 3, *p* < 0.001) and colour morph and study site (Wald Chi‐square = 12.5, *df* = 1, *p* < 0.001). There was no statistically significant interaction between developmental stage and colour morph (Wald Chi‐square = 7.3, *df* = 3, *p* = 0.063), indicating that the four developmental stages of the green and brown morphs use hosts relatively equally.

**Figure 5:**
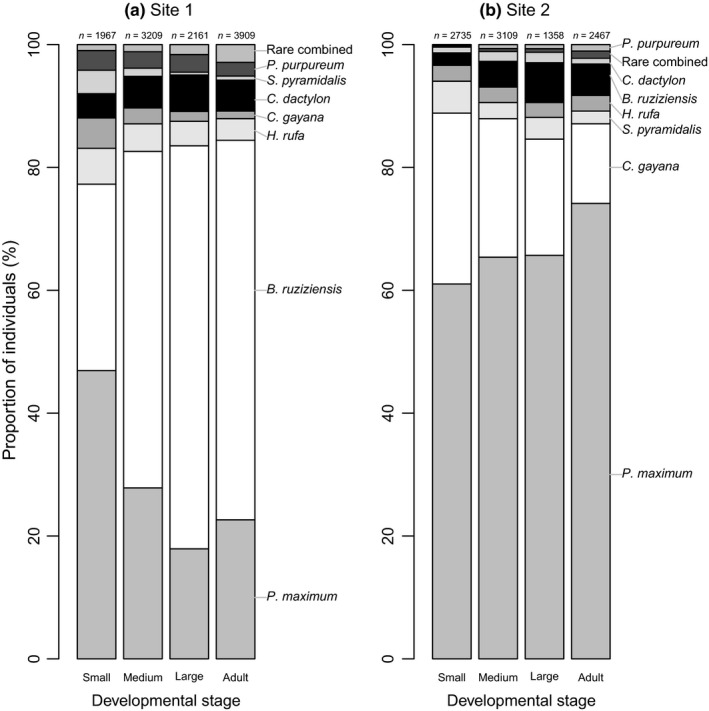
The proportion of observations on the seven most commonly used host plants (the rest of the grasses and sedges pooled as “Rare combined”) shown separately for the four developmental stages of *R. differens *at (a) Site 1 and (b) Site 2

The result of the generalized linear model suggests that different developmental stages used hosts differently, but this depended on the study site (Figure 5). At Site 1, small nymphs preferred the rare *P. maximum*, while older developmental stages shifted to the *B. ruziziensis*, which was the dominant grass of this site. Also, at Site 2, the dominant *P. maximum *was the most frequent host of the smallest nymphs, but it was an even more frequent host for older developmental stages (Figure 5). Overall, 22.5% of encountered *R. differens* comprised small nymphs, 30.2% medium nymphs, 16.8% large nymphs, and 30.5% adults.

**Figure 6:**
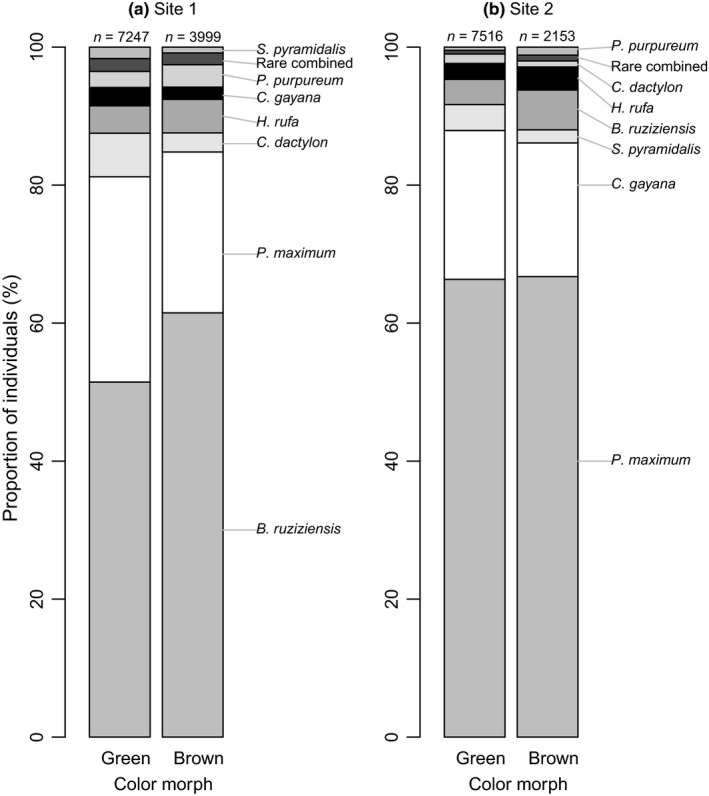
The proportion of observations on the seven most commonly used host plants (the rest of the grasses and sedges pooled as “Rare combined”) shown separately for green and brown morphs of *R. differens* at (a) Site 1 and (b) Site 2

The result of the generalized linear model also suggests that different colour morphs used hosts differently, but this depended on the study site (Figure 6). At both sites, proportionally more brown individuals were observed on *B. ruziziensis *than green individuals. Furthermore, at both Site 1 (64% of observations) and Site 2 (78%), green colour morphs were more common than brown morphs (Chi‐square goodness‐of‐fit tests; Site 1, χ^2^ = 938.1, *df* = 1, *p* < 0.001; Site 2, χ^2^ = 2974.6, *df* = 1, *p* < 0.001).

## 4 DISCUSSION

Our results show that, in the field, non‐swarming *R. differens* occur on a wide range of grass species, but obviously selectively. Availability of host plants seems to determine the pattern of host use in the field to some degree, since at both study sites the majority of *R. differens* were observed on the most common grass of the site. Yet, some host plants turned out to be more used than their abundance alone would predict. Selectivity in host plant use by *R. differens* has also been demonstrated experimentally (Valtonen et al., 2018). Among the common host plants found in our study, *R. differens* occurred more frequently on *P. maximum* than its cover alone would predict. Based on Opoke et al., (2018) (in press) (using the same dataset as here), the use of host plants also fluctuates seasonally, and during the greener seasons individuals use the more preferred host *P. maximum* more frequently. These findings suggest that *R. differens*’ preference for host plants can vary among habitats depending partly on host plant composition, but also on other factors, such as the physical and chemical qualities of the plants (reviewed in Bernays & Chapman, 1994), which could be important in the choice of host. In general, *R. differens* seems to be very opportunistic, potentially using a very wide range of host plant species.

A wide range of potential host plants for *R. differens* provides a good possibility for diet mixing (Bernays & Bright, 1993; Malinga et al., 2018b). The wide range of host plants utilized by *R. differens* has been observed earlier in wild populations (Bailey & McCrae, 1978; Swaine, 1964) and multiple‐choice experiments in the laboratory have shown that *R. differens* individuals feed on several host grasses when offered together (Valtonen et al., 2018). Diet mixing is associated with better performance in *R. differens*, including shorter developmental time and higher adult fresh weight and female fecundity (Malinga et al., 2018b). Diet mixing is considered to be beneficial for Orthopterans due to improvement in the balance of nutrients ingested or the dilution of toxins (Bernays & Minkenberg, 1997; Miura & Ohsaki, 2004; Unsicker et al., 2008).

It is evident that non‐swarming population of *R. differens* predominantly use grass inflorescences in the field (97% of feeding observations). This is also consistent with early observations made in East Africa (Bailey & McCrae, 1978, and references therein). Swarming *R. differens* are also occasionally recorded as pests on the developing seeds (at the milk stage) of several cereal crops (Swaine, 1964). In the laboratory, *R. differens* prefers grass inflorescences or seeds over stems or leaves (Hartley, 1967; Valtonen et al., 2018). *Ruspolia differens*’ nearly exclusive use of grass inflorescences and developing seeds as food possibly helps them to meet the energy requirements for their exceptional swarming behaviour (Bailey & McCrae, 1978). Flowers and grains are richer in protein, when compared to the leaves of grasses, and therefore generally more nutritious for herbivores (Bernays & Chapman, 1994; Roulston, Cane, & Buchmann, 2000). Thus, inflorescence and grain feeding may not only be necessary for survival, growth, and reproduction (Joern & Behmer, 1997) in non‐swarming populations but also for amassing the fat reserves required to maintain flight over considerable distances during swarming (Bailey & McCrae, 1978; Karuhize, 1972).

Female and male *R. differens* differ to some degree in their host choice (females were found more frequently on *B. ruziziensis* than males), possibly due to differences in nutritional requirements (Behmer & Joern, 1994; Boys, 1978; Unsicker et al., 2008), or this may reflect differential use of host plants for protection from predation. The sex ratio of *R. differens* was female‐biased, which could be mainly explained by the slower development of female nymphs (77% of large nymphs were females, as only females have the final 7th instar; Brits & Thornton, 1981). Furthermore, there could also be higher mortality of males in the large‐nymph stage. The slightly higher proportion of females among adults (56%) could be at least partly explained by higher recruitment rate of males to swarms, as swarms are typically male‐biased (Bailey & McCrae, 1978).

Also, we found that the four developmental stages of *R. differens* clearly differed in their choice of host plants, so that for the smallest nymphs, *P. maximum *is the preferred host, while increasingly older developmental stages were mostly found on the dominant grass of each site. Differences among developmental stages in host selection could be linked to the different level of protection different plant species can offer for different developmental stages from predators, through, e.g., plant architecture (Lawton, 1983). The different developmental stages might also vary in their nutritional requirements or physical capacity to use hosts (Hochuli, 2001; Werner & Gilliam, 1984). For example, young nymphs with small mandibles might be unable to consume tough hosts that adults can cope with (Hochuli, 2001). Also, feeding on tough grasses has been associated with large heads among Orthopterans (Bernays & Hamai, 1987).

Brown and green individuals differ slightly in their use of host plants. Overall, a larger proportion of brown individuals were observed on *B. ruziziensis*. Difference in use of host plants between colour morphs could be an adaptation of *R. differens* to optimise camouflage (Bailey & McCrae, 1978; Valverde & Schielzeth, 2015). However, for the strictly nocturnal nymphs, the role of colour morph in improving their survival in dim‐light conditions should be explored in further studies (see e.g., Meyer‐Rochow & Teh, 1991). The proportions of colour morphs are not static, as green morphs of *R. differens* increase in proportion after rainy seasons, when vegetation greens up (Matojo & Yarro, 2010; Opoke et al., 2018). The colour morph dynamic is a two‐way process, green nymphs are known to change to brown nymphs and vice versa during moulting (Robinson & Hartley, 1978), possibly induced by the changes in the environment.

Overall, the results of this study provide useful insights for the management and conservation of *R. differens*, which have hitherto been lacking. The wide range of host plants observed, selectivity in used plant species and their parts, and variability in host plant use among sexes, developmental stages, and colour morphs imply that diverse grass resources with continuous availability of inflorescences of host plants are necessary for maintaining harvestable populations of *R. differens*. The need for conserving habitats with such resources is more critical now than ever, considering the rapid degradation of critical grassland ecosystems in East Africa (Maitima et al., 2009), which could pose a threat to natural populations of this highly‐valued edible insect. Conservation practices such as rotational grazing and cultivation that allows host grasses to complete their life cycles, regulated bush burning, and gazetting hotspot habitats as conservation areas may boost wild populations of *R. differens*, and should be addressed in future studies. In captivity, it may be possible to successfully mass‐rear generations of *R. differens* based on diets formulated from host plants used by wild populations, or artificial feeds (Malinga et al., 2018a, 2018b).

In conclusion, this study has shown that in the field, non‐swarming *R. differens* mainly used the inflorescence of seven grass species, with an obvious bias for the most dominant grass species at each study site. However, *P. maximum* and *S. pyramidalis* were used more frequently than expected from their cover in the field. *Ruspolia differens* were observed predominantly feeding on inflorescences and much less frequently on leaves, stems, and inflorescence stalks. The results support our earlier finding that *R. differens* is a facultatively oligophagous grass‐specialist, but with different frequencies of host plants used among sexes, developmental stages, and colour morphs. *Panicum maximum* was the preferred host of the youngest nymphs of *R. differens*. These findings have important implications for the management and conservation of *R. differens*, implying that important hot spots of diverse host plants with continuous availability of inflorescence for *R. differens* should be a priority target for conservation in the face of current grassland degradation.

## CONFLICT OF INTERESTS

None declared.

## AUTHOR CONTRIBUTIONS

HR, PN, AV conceived research. RO contributed data. AV, PN, HR analysed data and conducted statistical analyses. RO, GMM, KR, PN, HR and AV wrote the manuscript. HR secured funding. All authors read and approved the manuscript.

## Data Availability

Data is available from the Dryad Digital Repository: https://doi.org/10.5061/dryad.f845h1n.
